# Effects of praziquantel on early life stages of Grass carp, *Ctenopharyngodon idella*

**DOI:** 10.17221/85/2024-VETMED

**Published:** 2025-03-24

**Authors:** Josef Velisek, Alzbeta Strouhova, Marie Sandova, Eliska Zuskova, Petr Dvorak, Alzbeta Stara

**Affiliations:** South Bohemian Research Centre of Aquaculture and Biodiversity of Hydrocenoses, Faculty of Fisheries and Protection of Waters, University of South Bohemia in Ceske Budejovice, Vodnany, Czech Republic

**Keywords:** antioxidant biomarkers, antiparasitic drug, carp, fish, histopathology

## Abstract

This study aimed to assess the toxicity of the anthelmintic drug praziquantel in the early life stages of grass carp (*Ctenopharyngodon idella*). The toxicity was evaluated based on mortality, early ontogeny development, growth, oxidative stress biomarkers, antioxidant enzymes and histopathology. Praziquantel at all tested concentrations (1, 2, 4 and 6 mg/l) showed no significant adverse effects on the hatching of grass carp. Concentrations of 2, 4 and 6 mg/l praziquantel caused significantly (*P < *0.01) higher mortality and slower growth compared with controls. Praziquantel at concentrations (4 and 6 mg/l) showed a significant (*P < *0.01) delay of early ontogeny of grass carp. Concentration of 2 mg/l praziquantel caused significantly (*P < *0.01) higher GST activity than controls. Among the groups, no histological changes were detected in tissues. For the early life of grass carp, praziquantel is safe at concentrations ≤1 mg/l.

With the growth of aquaculture intensification comes the challenge of managing fish health, particularly controlling parasitic infections, which can severely impact fish populations. One of the common parasites affecting freshwater fish is the trematode, which is responsible for diseases that can lead to high mortality rates if left untreated. Drugs are commonly applied to control trematodes, and some traditional parasiticidal drugs such as praziquantel, fenbendazole, levamisole hydrochloride, and ivermectin have been used for decades (Kolarova et al. 2022).

Praziquantel [2-(cyclohexylcarbonyl)-1,2,3,6,7, 11b-hexahydro-4H-pyrazino (2,1-a) isoquinolin-4-one] is an anthelmintic drug that reduces cestodes and trematodes in fish when injected, fed, or used as a water bath treatment (Bader et al. 2019). Praziquantel has lacked registration for its use in aquaculture thus its use was only possible in the „off-label“ cascade manner regulated by Council Directive 90/676/EEC, Directive 2001/82/EC and Commission Regulation No. 37/2010. Nowadays, the maximum residual limit of praziquantel and its isomers in muscle and skin in natural proportions was set at 20 μg/kg for fin fish in the EU legislation [Commission Regulation (EU) No. 37/2010]. In veterinary medicine, one of the most commonly used agents with anti-flatworm activity is praziquantel; yet, no praziquantel products are labelled for use in fish in the United States. Veterinarians may use praziquantel preparations approved for other vertebrate species under the Animal Medicinal Drug Use Clarification Act (AMDUCA) (Bader et al. 2019). Recommended concentrations of therapeutic baths are from 0.25 mg/l to 50 mg/l. Oral praziquantel administration for single doses ranges from 50 mg/l to 200 mg/l, and for repeated multiple doses, ranges from 7 mg/l to 75 mg/l. Appropriate dosage depends on the fish species, age, size, and specific environmental conditions in which the fish are kept (Bader et al. 2019).

In aquaculture, praziquantel has emerged as a critical tool for managing parasitic infestations in fish. However, using praziquantel (PZQ) in fish farming raises concerns regarding its safety, efficacy, and environmental impact. Despite its effectiveness, the effect of PZQ on fish, particularly at varying dosages and exposure durations, requires thorough investigation to ensure its safe application in aquaculture practices. Little information is available on the toxicity of praziquantel to fish, and the safety margin between a treatment rate and toxic doses is unknown for most fish species. For grass carp weighing 9.1 g, the 24 h and 96hLC50 concentrations of PZQ are 63.4 and 60.6 mg/l, and the 24 h and 96hLC0 concentrations are 60.0 and 60.0 mg/l (Mitchell and Hobbs 2007). PZQ generally has a wide margin of safety and relatively few side effects in mammals (Andrews et al. 1983). Previous studies have highlighted potential adverse effects of PZQ on fish, including stress responses, changes in metabolic activity, and histopathological alterations (Soltanian et al. 2018; Zuskova et al. 2018; Velisek et al. 2022). Depending on species, developmental stages, and environmental conditions, these effects may vary significantly. However, scientific sources lack data on the particular impact of PZQ on the embryo and larvae of grass carp. Therefore, it is crucial to understand the specific effects of praziquantel on different fish species to optimise treatment protocols and minimise potential risks.

This study aims to explore the effects of PZQ on the embryo and larvae of grass carp, commonly found in aquaculture. By evaluating indicators such as mortality, growth rate, ontogenetic development, and physiological responses (oxidative stress and antioxidant biomarkers), we seek to provide a comprehensive assessment of praziquantel’s impact. Additionally, we investigated the histopathological changes in grass carp tissues post-treatment to gain insights into any sub-lethal effects that may not be immediately apparent.

## MATERIAL AND METHODS

### Chemicals and chemical analysis

Praziquantel from Ecological Laboratories Inc., USA, was used for our test. Concentrations of praziquantel were checked daily before and after the bath renewal by ultrahigh-performance liquid chromatography (UHPLC) using the method of Zrncic et al. (2014). The actual concentration values did not differ from the nominal concentration by more than ±1%.

### Experimental protocol

Fertilised grass carp eggs were obtained from a hatchery of the University of South Bohemia in Ceske Budejovice, Faculty of Fisheries and Protection of Waters, Czech Republic.

The investigation was conducted using the Organization for Economic Cooperation and Development Guidelines for Testing of Chemicals No. 210. Ten hours post fertilisation, 100 fertilised grass carp eggs were placed into each of eighteen glass basins with the praziquantel solution. Each experimental condition was triplicated once, and a total of 1 800 fertilised eggs of grass carp were used. The concentrations of PZQ used were:

1. E1 group – 1.0 mg/l;
2. E2 group – 2.0 mg/l [concentrations for antiparasitic bath; Noga (2010)];
3. E3 group – 4.0 mg/l;
4. E4 group – 6.0 mg/l;
5. C control group – dechlorinated water only;
6. E-C ethanol group – (0.5 ml/l) was used for the highest concentration of the tested substance. Ethanol was used as a solvent due to the low solubility of tested substances in water.

The solution for each group was renewed daily. Mortality, morphological anomalies, oxygen saturation, and pH were monitored daily, and dead grass carp were removed. Water quality parameters were as follows: temperature 22.1 ± 0.6 °C, dissolved oxygen saturation 95%, pH 7.82–8.01, acid neutralisation capacity (ANC4.5) 0.57 mmol/l and chemical oxygen demand 0.81 mg/l. From day 4, larvae of grass carp were fed *ad libitum* with *Artemia salina nauplii.* On days 8, 15, 22, and 29, six early life stages of grass carp in each experimental group were collected to examine ontogenetic development and growth. The toxicity test was terminated after 29 days when the tested fish were sampled for biochemical and histopathological analyses (only groups C, E-C, E1 and E2; groups E3 and E4 died during the experiment). Before sampling, fish were euthanised (MS222, 250 mg/l), weighed and stored in tubes at –80 °C until further analyses. Six juvenile grass carp from each survivor group were placed in 10% formalin for histopathological analyses.

This study was conducted in compliance with the Czech Republic regulations 166/1996 and 246/1992 and approved by the Departmental Expert Committee for Authorisation of Experimental Projects of the Ministry of Education, Youth, and Sports of the Czech Republic (Permit No. MSMT-3126/2021-3).

### Early ontogeny

On days 8, 15, 22, and 29, six early life stages of grass carp in each experimental group were collected for examination of the ontogenetic development period. Developmental periods were defined according to Yi et al. (2006), who described thirty embryonic stages (E1–E30), eighteen post-hatch stages (P1–P18), and one juvenile stage (J1) of grass carp.

### Growth rate

On days 8, 15, 22, and 29, six grass carp in each experimental group were collected to examine growth. The total length (TL) was measured by stereomicroscope using a micrometre. Mass was measured (0.1 mg) with a Mettler-Toledo scale.

The mean specific growth rates (SGR) of experimental groups were calculated for the period from day 8 (the first sampling day) to day 29 (end of the test) using the method described by OECD (2000).

### Oxidative stress and antioxidant biomarkers

Biomarkers were evaluated in the surviving grass carp of groups (only groups C, E-C, E1 and E2) after 29 days of exposure.

Whole-body samples were immediately frozen and stored at –80 °C for analysis. Frozen samples were weighed and homogenised (1 : 10, w/v) with a ball homogenizer (TissueLyser II; QIAGEN^®^, Hilden, Germany) using 50 mM potassium phosphate buffer, pH 7.0, containing 0.5 mM EDTA according to methods Stara et al. (2021). The homogenates were centrifuged at 4 °C in a refrigerated centrifuge (Beckman Optima L-90 K Ultracentrifuge; Brea, USA) at 12 000 *g* for 30 min for superoxide dismutase (SOD) and catalase (CAT) assays; at 4 000 *g* for 15 min for glutathione *S*-transferase (GST) and reduced glutathione (GSH) measurements and at 5 000 *g* for 30 min for the acetylcholinesterase activity (AChE) assay. Homogenates used for the lipid peroxidation (LPO) assay and total protein level determination were analysed without centrifugation.

LPO activity was measured using the TBARS (thiobarbituric acid reactive substances) assay proposed by Lushchak et al. (2005). SOD activity was determinated using the method of Marklund and Marklund (1974). For the CAT activity assay, the method of Beers and Sizer (1952) was used. GST activity was measured using the method of Habig et al. (1974). GSH levels were assessed using Tipple and Rogers’s (2012) method. AChE activity was recovered by the methods described by Ellman et al. (1961).

### Histology

Histological examination was conducted in experimental groups after 29 days of exposure.

Six juvenile grass carp from each survivor treatment were placed in 10% buffered formalin, and after 24 h the fish were transferred into 70% ethyl alcohol.

Fixed samples were prepared with standard histological techniques, dehydrated, embedded in paraffin, cut on a rotary microtome, stained with haematoxylin and eosin, and examined by light microscope combined with camera system type E-600 (Olympus BX51; Tokyo, Japan).

### Statistical analysis

Differences in cumulative mortality between treatments were assessed using contingency tables (χ^2^). Results were tested using the variance analysis software Statistica v14.0 (StatSoft, Czech Republic) (one-way ANOVA – Unequal N HSD Test).

## RESULTS AND DISCUSSION

### Mortality, hatching

**Figure 1:**
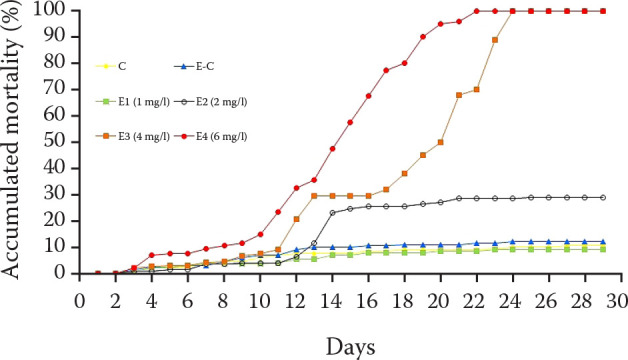
Accumulated percentage mortality of grass carp (*Ctenopharyngodon idella*) during 29 days of praziquantel exposure C = control group; E1–4 = concentrations of PZQ; E-C = ethanol group; PZQ = praziquantel

Some studies have reported that the early life stages of fish are highly sensitive to chemicals (Woltering 1984; Chromcova et al. 2015; Plhalova et al. 2018). While we have information on the toxicity of praziquantel to fish, we do not know of its effect on the embryo and larvae of grass carp. This gap in knowledge highlights the need for focused research on the impact of praziquantel on grass carp’s early life stages. Understanding the potential risks is crucial for managing both aquaculture practices and wild populations, ensuring that chemical treatments do not adversely affect fish development and survival during these critical early stages (Merola et al. 2022). In our experiment, the eggs in all groups hatched by day 2. No significant adverse effects of PZQ on hatching were observed. Significant (*P* < 0.01) differences in total accumulated mortality were found in grass carp exposed to the PZQ concentrations 2, 4 and 6 mg/l compared with controls (Figure 1). All grass carp from group E4 died within 21 days of exposure, and group E3 within 24 days. At the end of the test, accumulated mortality in the group exposed to PZQ in concentrations of 1 mg/l (E1), 2 mg/l (E2), 4 mg/l (E3) and 6 mg/l (E4) was 9, 29, 100 and 100%. In ethanol control (E-C), control (C) was 12 and 10.5 %, respectively.

PZQ is toxic to fish. The 24hLC50 for grass carp is 63.4 mg/l (Mitchell and Hobbs 2007), and 96hLC50 for barbel (*Barbus barbus*) is 28.6 mg/l PZQ (Zuskova et al. 2018). In our study, longer exposure time (29 days) caused higher mortality.

### Early ontogeny

**Figure 2:**
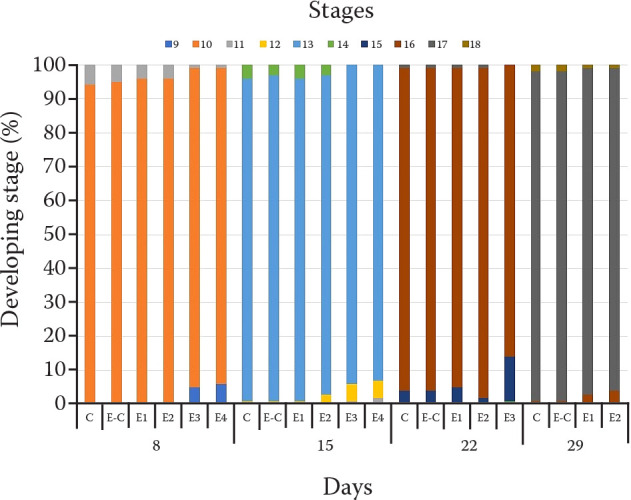
Figure 2. Developing stages of grass carp (*Ctenopharyngodon idella*) during 29 days of praziquantel exposure (9) Yolk-absorption stage; (10) Dorsal-fin-differentiation stage; (11) Notochord-tip-lifting stage; (12) Two-chamber-gas-bladder stage; (13) Pelvic-fin-bud stage; (14) Dorsal-fin-formation stage; (15) Anal-fin-formation stage; (16) Pelvic-fin-formation stage; (17) Squamation stage; (18) Juvenile stage

Delayed early development is a common chronic toxicity response observed in fish. This sensitivity to toxicants during early development has been well-documented in several studies (Velisek and Stara 2018; Islam et al. 2019; Velisek et al. 2022). In our research, from 8 days, PZQ at concentrations 4 and 6 mg/l showed a significant (*P* < 0.01) delay of early ontogeny of grass carp (Figure 2). The delayed development of grass carp exposed to praziquantel can be attributed to developmental events such as organogenesis. On the other hand, Velisek et al. (2022) reported no delay in the early life stages of the common carp’s after PZQ exposure.

C = control group; E1–4 = concentrations of PZQ; E-C = ethanol group; PZQ = praziquantel

Changes in early ontogenetic development are described mainly after exposure to pesticides (Velisek and Stara 2018) and human drugs (Sehonova et al. 2017).

### Growth

**Figure 3:**
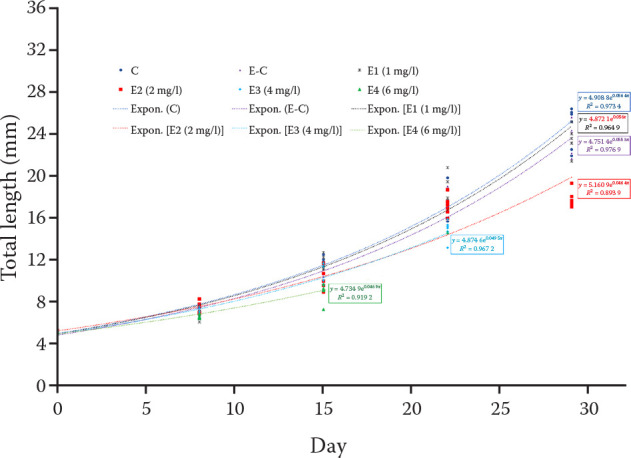
The total length of grass carp (*Ctenopharyngodon idella*) during 29 days of praziquantel exposure C = control group; E1–4 = concentrations of PZQ; E-C = ethanol group; PZQ = praziquantel

**Figure 4:**
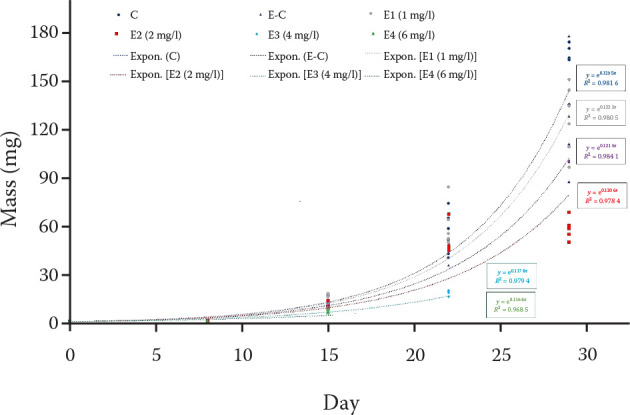
The weight of grass carp (*Ctenopharyngodon idella*) during 29 days of praziquantel exposure C = control group; E1–4 = concentrations of PZQ; E-C = ethanol group; PZQ = praziquantel

Generally, stress conditions such as polluted aquatic environments and diseases result in decreased fish growth. Praziquantel, a commonly used antiparasitic drug, can also affect fish growth. The concentrations of praziquantel play a crucial role in its effects on fish. Therefore, it is important to carefully monitor and regulate the dosage of PZQ to minimise its negative impacts on fish growth and health (Noga 2010). Appropriate dosage depends on the fish species, age, size, and specific environmental conditions in which the fish are kept. In our test, beginning on day 8 of exposure, grass carp in group E4 (6 mg/l) showed significantly (*P* < 0.01) lower total length (Figure 3) and mass (Figure 4) compared with controls. Beginning on day 15 of exposure, grass carp in groups E2 (2 mg/l) and E3 (4 mg/l) also showed significantly (*P* < 0.01) lower total length and mass compared with control. Velisek et al. (2022) reported a slow-down in growth of the early life stages of common carp after PZQ exposure at concentrations 3 and 4 mg/l. The Fulton’s weight condition factor values of grass carp are given in Table 1. At the end of the experiment (29 days), the FCF values were significantly (*P* < 0.01) lower in group E2 (2 mg/l) compared with controls. Compared to the control, inhibition of growth grass carp was 2.39 and 22.45% in groups E1 and E2, respectively (Table 1).

**Table 1:** Growth of grass carp during 29 days of exposure to praziquantel

Group	C	E-C	Praziquantel			
			E1 (1 mg/l)	E2 (2 mg/l)	E3 (4 mg/l)	E4 (6 mg/l)
m_8_	1.43 ± 0.19	1.28 ± 0.42	1.30 ± 0.21	1.18 ± 0.20	1.13 ± 0.27	1.10 ±0.15
m_29_	139.60 ± 16.26	101.09 ± 26.11	129.40 ± 20.81	55.55 ± 10.75*	†	†
FWC	1.10 ± 0.09	0.91 ± 0.13	0.97 ± 0.10	0.86 ± 0.08*	†	†
SGR	21.76	19.90	21.24	16.86	†	†
I (%)	–	8.55	2.39	22.45	†	†

*Significant (*P* < 0.01) difference of experimental groups compared to the control group (one-way ANOVA); ^†^Data are not given, because all fish in the groups died during the experiment

C = control group; E1–4 = concentrations of PZQ; E-C = ethanol group; FWC = mean Fulton’s condition factor of fish after 29 days exposure; I = inhibition of specific growth in selected group after 21 days exposure; m_8_, m_29_ = mean carp weight in group after 8 and 29 days exposure (mean ± SD, mg); PZQ = praziquantel; SD = standard deviation; SGR = specific growth rate in group after 29 days exposure

### Oxidative stress and antioxidant response

Oxidative stress has been defined as an imbalance of oxidants and antioxidants favouring the oxidants that can evoked by pollutants and chemicals, potentially leading to cell damage. In our test, grass carp exposed to the PZQ concentration of 2 mg/l showed significantly (*P* < 0.01) higher GST activity compared to the controls (Table 2). No significant differences among groups were seen in TBARS, SOD, CAT, GSH, or AChE activity. In our study, the elevation of GST activity in the whole-body homogenate of praziquantel-exposed grass carp indicates that the antioxidant system seemed to maintain a balance of production and scavenging ROS and prevented oxidative damage to tissues. Velisek et al. (2022) reported that exposure to praziquantel at concentrations 3 and 4 mg/l decreased SOD and CAT activity in the whole-body homogenate of early life stages of common carp. PZQ at concentrations 10 and 20 mg/l affected the activity of CAT, SOD, GR, and GST as well as levels of GSH in the liver and muscle of the barbel (Zuskova et al. 2018). Changes in antioxidant enzymes were observed in the Kolarova et al. (2022) study after a therapeutic bath with levamisole, fenbendazole, and ivermectin in common carp.

**Table 2:** Oxidative stress biomarker and antioxidant enzymes in the homogenate of grass carp after praziquantel exposure

Group	C	E-C	Praziquantel	
			E1 (1 mg/l)	E2 (2 mg/l)
TBARS (nmol/mg protein)	1.062 ± 0.225	0.882 ± 0.245	1.393 ± 0.666	0.904 ± 0.105
SOD (nmol NBT/min/mg protein)	0.243 ± 0.037	0.287 ± 0.048	0.319 ± 0.100	0.327 ± 0.054
CAT (μmol H_2_O_2_/min/mg protein)	0.003 ± 0.001	0.004 ± 0.001	0.003 ± 0.001	0.006 ± 0.004
GST (nmol/min/mg protein)	0.005 ± 0.004	0.009 ± 0.008	0.018 ± 0.016	0.049 ± 0.014*
GSH (nmol GSH/mg protein)	5.371 ± 1.357	4.528 ± 1.083	3.649 ± 0.987	4.898 ± 0.867
AChE (nmol/min/mg protein)	0.949 ± 0.249	1.390 ± 0.389	0.795 ± 0.252	1.108 ± 0.535

*Significant (*P* < 0.01) difference of experimental groups compared to the control group

AchE = acetylcholinesterase activity; C = control group; CAT = catalase; E1–2 = concentrations of PZQ; E-C = ethanol group; GSH = reduced glutathione; GST = glutathione *S*-transferase; PZQ = praziquantel; SOD = superoxide dismutase; TBARS = thiobarbituric acid reactive substances

### Morphological abnormalities and histology

Our study observed no significant differences in the type and occurrence of morphological abnormalities in tested grass carp during the test. Similarly, Velisek et al. (2022) did not find morphological abnormalities in the embryos and larvae of common carp after PZQ exposure. Morphological abnormalities and body deformations in the early life stages of fish are mainly observed after human drugs (Van den Brandhof and Montforts 2010; Porretti et al. 2022) and pesticides (Velisek and Stara 2018; Islam et al. 2019; Sharma et al. 2021).

No histological changes were demonstrated in the tissues (gills, skin, kidney, hepatopancreas, and intestine) after PZQ exposure. Velisek et al. (2022) reported extensive steatosis of the hepatopancreas attributed to ethanol in juvenile carp; however, grass carp from our test did not show such a high sensitivity to the similar amount of ethanol used as the praziquantel solvent.

Many therapeutic techniques and treatments developed and used in mammals require validation before being considered safe and effective in aquatic species. This study aimed to evaluate the effects of praziquantel on the early life stages of grass carp. The results demonstrated that exposure to praziquantel induced a delay in ontogenetic development, significantly higher mortality, increased glutathione *S*-transferase activity, and reduced growth in the early life stages of grass carp. Despite these adverse effects, praziquantel was found to be safe for the early life stages of grass carp at concentrations ≤1 mg/l. This study underscores the importance of thorough evaluation and species-specific studies in the development and application of therapeutic treatments in aquaculture.

## References

[R1] Andrews P, Thomas H, Pohlke R, Seubert J. Praziquantel. Med Res Rev. 1983 Apr-Jun;3(2):147-200. 6408323 10.1002/med.2610030204

[R2] Bader C, Starling DE, Jones DE, Brewer MT. Use of praziquantel to control platyhelminth parasites of fish. J Vet Pharmacol Ther. 2019 Mar;42(2):139-53. 30556228 10.1111/jvp.12735

[R3] Beers RF, Sizer IW. A spectrophotometric method for measuring the breakdown of hydrogen peroxide by catalase. J Biol Chem. 1952 Mar;195(1):133-40. 14938361

[R4] Chromcova L, Blahova J, Zivna D, Plhalova L, Casuscelli F, Divisova L, Prokes M, Faggio C, Tichy F, Svobodova Z. NeemAzal TS toxicity to early-life stages of common carp Cyprinus carpio L. Vet Med-Czech. 2015 Jan;60(1):23-30.

[R5] Ellman GL, Courtney KD, Andres V, Featherstone RM. A new and rapid colorimetric determination of acetylcholinesterase activity. Biochem Pharmacol. 1961 Jul;7:88-95. 13726518 10.1016/0006-2952(61)90145-9

[R6] Habig WH, Pabst MJ, Jakoby WB. Glutathione S-transferases. First enzymatic step in mercapturic acid formation. J Biol Chem. 1974 Nov;249(22):7130-9. 4436300

[R7] Islam MA, Hossen MS, Sumon KA, Rahman MM. Acute toxicity of imidacloprid on the developmental stages of common carp. Toxicol Environ Health Sci. 2019 Oct;11:244-51.

[R8] Kolarova J, Zuskova E, Velisek J. Efficacy of therapeutic bath with selected antiparasitic drugs on a Dactylogyrus anchoratus infection in juvenile common carp (Cyprinus carpio). Vet Med-Czech. 2022 Nov;67(12):620-7. 10.17221/66/2022-VETMED PMC11154880 38845786

[R9] Lushchak VI, Bagnyukova TV, Husak VV, Luzhna LI, Lushchak OV, Storey KB. Hyperoxia results in transient oxidative stress and an adaptive response by antioxidant enzymes in goldfish tissues. Int J Biochem Cell Biol. 2005 Aug;37(8):1670-80. 15896673 10.1016/j.biocel.2005.02.024

[R10] Marklund S, Marklund G. Involvement of superoxide anion radical in autoxidation of pyrogallol and a convenient assay for superoxide dismutase. Eur J Biochem. 1974 Sep;47(3):469-74. 4215654 10.1111/j.1432-1033.1974.tb03714.x

[R11] Merola C, Fabrello J, Matozzo V, Faggio C, Iannetta A, Tinelli A, Crescenzo G, Amorena M, Perugini M. Dinitroaniline herbicide pendimethalin affects development and induces biochemical and histological alterations in zebrafish early-life stages. Sci Total Environ. 2022;828:154414. 35278537 10.1016/j.scitotenv.2022.154414

[R12] Mitchell AJ, Hobbs MS. The acute toxicity of praziquantel to grass carp and golden shiners. N Am J Aquacult. 2007 Jan;69(3):203-6.

[R13] Noga EJ. Fish disease: Diagnosis and treatment. Ames: Wiley-Blackwell; 2010. 519 p.

[R14] OECD – Organization for Economic Cooperation and Development. Guideline for testing of chemicals 215. Fish juvenile growth test. Paris: OECD; 2000. 16 p.

[R15] Plhalova L, Blahova J, Divisova L, Enevova V, Faggio C, Tichy F, Vecerek V, Svobodova Z. The effects of subchronic exposure to NeemAzal T/S on zebrafish. Chem Ecol. 2018 Feb;34:199-210.

[R16] Porretti M, Arrigo F, Di Bella G, Faggio C. Impact of pharmaceutical products on zebrafish: An effective tool to assess aquatic pollution. Comp Biochem Physiol C. 2022 Nov;261:109439. 10.1016/j.cbpc.2022.109439 35961532

[R17] Sehonova P, Plhalova L, Blahova J, Doubkova V, Tichy F, Fiorino E, Faggio C, Svobodova Z. Toxicity of naproxen sodium and its mixture with tramadol hydrochloride on fish early life stages. Chemosphere. 2017 Dec;188:414-23. 28898774 10.1016/j.chemosphere.2017.08.151

[R18] Sharma S, Iqbal Dar O, Singh K, Kaur A, Faggio C. Triclosan elicited biochemical and transcriptomic alterations in Labeo rohita larvae. Environ Toxicol Pharmacol. 2021 Nov; 88:103748. 10.1016/j.etap.2021.103748 34534692

[R19] Soltanian S, Vazirzadeh A, Akbary P. Effect of praziquantel on hemato-immunological indices in common carp. Iran J Sci Technol Trans Sci. 2018 Mar;42:1015-25.

[R20] Stara A, Pagano M, Albano M, Di Bella G, Albergamo A, Koutkova Z, Sandova M, Velisek J, Matozzo V, Faggio C. Effects of long-term exposure of Mytilus galloprovincialis to thiacloprid: A multibiomarker approach. Environ Pollut. 2021 Nov;289:117892. 34385134 10.1016/j.envpol.2021.117892

[R21] Tipple TE, Rogers LK. Methods for the determination of plasma or tissue glutathione levels. Meth Mol Biol. 2012 Jan;889:315-24. 10.1007/978-1-61779-867-2_20 PMC3680121 22669674

[R22] Van den Brandhof EJ, Montforts M. Fish embryo toxicity of carbamazepine, diclofenac and metoprolol. Ecotoxicol Environ Saf. 2010 Nov;73(8):1862-6. 20832863 10.1016/j.ecoenv.2010.08.031

[R23] Velisek J, Zuskova E, Kubec J, Sandova M, Stara A. Effects of praziquantel on common carp embryos and larvae. Sci Rep. 2022 Oct;12(1):17290. 36241766 10.1038/s41598-022-21679-2 PMC9568519

[R24] Velisek J, Stara A. Effect of thiacloprid on early life stages of common carp (Cyprinus carpio). Chemosphere. 2018 Mar;194:481-7. 29232641 10.1016/j.chemosphere.2017.11.176

[R25] Woltering DM. The growth response in fish chronic and early life stage toxicity tests: A critical review. Aquat Toxicol. 1984 Feb;5(1):1-21.

[R26] Yi B, Liang Z, Lin R, He M. A study of the early development of grass carp, black carp, silver carp, and bighead carp in the Yangtze River, China. In: Chapman DC, editor. Early development of four cyprinids native to the Yangtze River, China. U.S. Geological Survey Data Series 239; 2006. p. 11-51.

[R27] Zuskova E, Piackova V, Valentova O, Zalohova K, Velisek J. Praziquantel toxicity to fish Danio rerio and planktonic crustacean Daphnia magna. Vet Med-Czech. 2022 Oct;67(11):579-84. 10.17221/7/2022-VETMED PMC11016302 38623477

[R28] Zuskova E, Piackova V, Machova J, Chupani L, Steinbach C, Stara A, Velisek J. Efficacy and toxicity of praziquantel in helminth-infected barbel. J Fish Dis. 2018 Apr;41(4): 643-9. 29349797 10.1111/jfd.12764

[R29] Zrncic M, Gros M, Babic S, Kastelan-Macan M, Barcelo D, Petrovic M. Analysis of anthelmintics in surface water by ultra high performance liquid chromatography coupled to quadrupole linear ion trap tandem mass spectrometry. Chemosphere. 2014 Mar;99:224-32. 24289978 10.1016/j.chemosphere.2013.10.091

